# Cyclododecane shaping, sublimation rate and residue analysis for the extraction of painting micro-samples from resin cross-sections

**DOI:** 10.1038/s41598-022-22448-x

**Published:** 2022-11-16

**Authors:** Victory Armida Janine Jaques, Eva Zikmundová, Jiří Holas, Tomáš Zikmund, Jozef Kaiser, Katarína Holcová

**Affiliations:** 1 Faculty of Science, Institute of Geology and Palaeontology, Charles University, Albertov 6, 12843 Praha, Czech Republic; 2 CEITEC - Central European Institute of Technology, Brno University of Technology, Purkyňova 123, 612 00 Brno, Czech Republic

**Keywords:** Techniques and instrumentation, Materials science

## Abstract

Cross-section preparation of painting micro-samples is part of their routine analysis. This type of preparation can be used for several analytical techniques, such as scanning electron microscopy, Fourier-transform infrared spectroscopy, Raman spectroscopy, and optical microscopy. These techniques offer high-resolution imaging and/or elemental information, providing access to technical and material data important for the interpretation, preservation, and restoration of painted artworks. However, it also means that the material from the sample embedded in the resin becomes unreachable for further analysis, except for the polished surface of the cross-section. Degradation of the embedding medium can also occur over time, which can lead to misinterpretation, loss of information, or even complete destruction of the embedded sample. In the field of cultural heritage, cyclododecane (CDD) is commonly used for the consolidation and protection of objects, and is used in the preparation of cross-sections to prevent contamination of the sample by the embedding medium. This study enhanced the existing preparation process by shaping the CDD layer to enable extraction of the micro-sample from the resin if needed, without compromising the integrity of the sample. Moreover, the purity, the sublimation rate in a normal environment and a vacuum, and the impact of CDD on three different types of samples (historical painting on a canvas, wall painting fragment, model sample) were examined.

## Introduction

Painting micro-samples, along with many other cultural heritage-related objects, are usually brittle, sensitive, of mixed composition (organic and inorganic) and unique. The cultural heritage micro-samples analysed in the laboratory are usually very small (< 3 mm) and their non-reproducibility makes them rare and important pieces of history that should remain as intact as possible.

The painting technique encountered in the micro-samples strongly varies from a single to several coloured layers, which are often thinner than 10 µm each^1^ and made of diverse materials. These paint layers are applied on various supports, sometimes with a protective layer on top^2^. The sequence of layers is called a stratigraphy of the micro-sample, which can be observed and analysed as a cross-section. Its analysis gives valuable information on the painting technique, the type of used materials, the porosity, the degradation, or the interaction of the components. The gained information is essential for the appropriate preservation and restoration of the artworks^3^. However, once prepared as a cross-section, the three-dimensional (3D) information hidden under the polished surface of the cross-section cannot be accessed anymore.

Cross-section preparations vary depending on the required analyses^4^. Scanning electron microscopy (SEM)^2,5–13^, Fourier-transform infrared spectroscopy (FTIR)^8,9,14^, Raman spectroscopy (RS)^9,15,16^ and optical microscopy (OM)^1,4,17^ are the most common techniques used in the field. While a combination of analysis offers more insightful investigation^4,18–22^.

Three common embedding techniques are used to prepare cross-sections: flat embedding^23^, slotted-capsulated block^24^, and capsule embedding^25^. Khandekar^26^ clearly described and compared these embeddings. Several mediums can be used for embeddings, depending on the required analysis^27^. The most common for SEM measurements is a resin^28,29^, while diamond-cell, potassium bromide (KBr) or sodium chloride (NaCl) are used for FTIR embedding along with the resin^27,30–35^.

Different types of resin are available and used in the field^36^. Their properties vary and must be adapted to the required analysis and sample type, which means the cross-section has limited use. The specific properties of resins cover hot/cold preparation, curing time and temperature^37,38^, transparency^39^, colour, fluorescence^40^, hardness, edge retention, and viscosity influencing the infiltration^36^. When in contact with the sample, the resin has an impact on it. This impact can be positive, such as a consolidation of the sample, or negative when morphological or chemical changes develop^41,42^. Morphological changes occur because of the resin shrinkage while curing^42,43^, or due to the dissolution/recrystallisation of the material of the sample itself after the interaction with some resin components, creating artefacts^36^. Although some reactions can be negligible, with no previous analysis, the difference between the original state and the state after the sample preparation might be overlooked and lead to wrong interpretations. This happens when a low viscosity resin penetrates the micro-sample^36^.

Different types of resin, various preparations of cross-sections, or the use of protective material around the sample have been tested and described by several authors^14,27,44^. Cyclododecane (CDD) is often used as a temporary consolidant^45,46^ and/or as a protective layer^47,48^ for the samples. New uses are also being discovered, such as its contrast enhancement^49,50^, which is helpful in the cultural heritage field^44,51^. Menthol is also often mentioned to be used for the same purpose, but it is still in the research phase^52,53^. Both chemicals can cause health issues, and should be handled carefully^54,55^.

Cyclododecane is a saturated cyclic alkane (C12H24)^56–58^, a waxy solid^51^, highly hydrophobic and chemically stable, melting between 58 and 61 °C^55^. It can be used pure and melted^59^, between 80 and 100 °C^60^ or mixed with solvents^47,61^. Depending on the method and the temperature of application and cooling^62^, the recrystallisation is affected^63^ and affects the CDD properties^47^, such as the penetration depth, film thickness^63^ and sublimation rate^55,64^. Prescutti et al.^59^ determined that a highly concentrated CDD solution or pure melted CDD has a low infiltration power and recrystallises more quickly compared with a low-concentration solution in large crystals, creating a thick and compact layer on the surface of the sample.

CDD normally sublimes in approximately 24 h at room temperature^55,65^, but as was already mentioned, the application process, the layer thickness and the storage environment impact the sublimation time^55,64,66^. Thus, sublimation can be a matter of hours to weeks^67^. In her thesis, Piotrowski^67^ thoroughly explained that the sublimation rate of CDD depends on the application methods. Hence, the process can be deliberately sped up or slowed down. According to several studies, CDD sublimes leave no trace, as they are not detectable and do not modify the structure of the sample^44,55,68,69^. However, different studies suggest otherwise^70^ with Papini et al.^71^ finding that melted CDD released more free fatty acids from oil paintings depending on their age than CDD in solution, which could interfere with the study of organic oil binders. However, the dissolution of the oil in CDD could occur due to the application temperature of the melted CDD, rather than the application of CDD itself^71^.

Any embedding medium has an impact on the sample, which can appear as various artefacts, such as swelling or recrystallisation. Moreover, the part of the sample embedded in the resin becomes unreachable, which hinders further measurements and therefore gains information from it. Finally, uncertainty about the embedding medium degradation over time can be an issue for later microscopic analysis, leading to misinterpretation or significant alterations of the sample. The issue of micro-sample contamination by the embedding medium has already been addressed using a CDD protective layer. Our study, however, aims to enhance the widely used preparation technique of cross-sections by exploring the feasibility of micro-sample extraction using the sublimation of CDD, and addressing the other issues consequently.

Common materials, i.e., resin as an embedding medium and CDD as a protective layer, are used to make the extraction technique accessible for all laboratories without extra equipment. A suitable application process of the CDD enables a later extraction, which was examined, together with other properties of the CDD. Moreover, as the cross-sections are routinely analysed in a low-vacuum SEM, the sublimation rate of CDD under different pressures was investigated.

## Materials and methods

### Samples

Three different micro-samples, representing most common paintings regarding their materials, size, brittleness, and porosity, were chosen for this study: a painting on canvas support, a wall painting, and a painting on a wooden support. The first two samples are real, i.e. historical samples (RS), and the wooden painting is a model sample (MS) created by a restorer.

**Figure 1:**
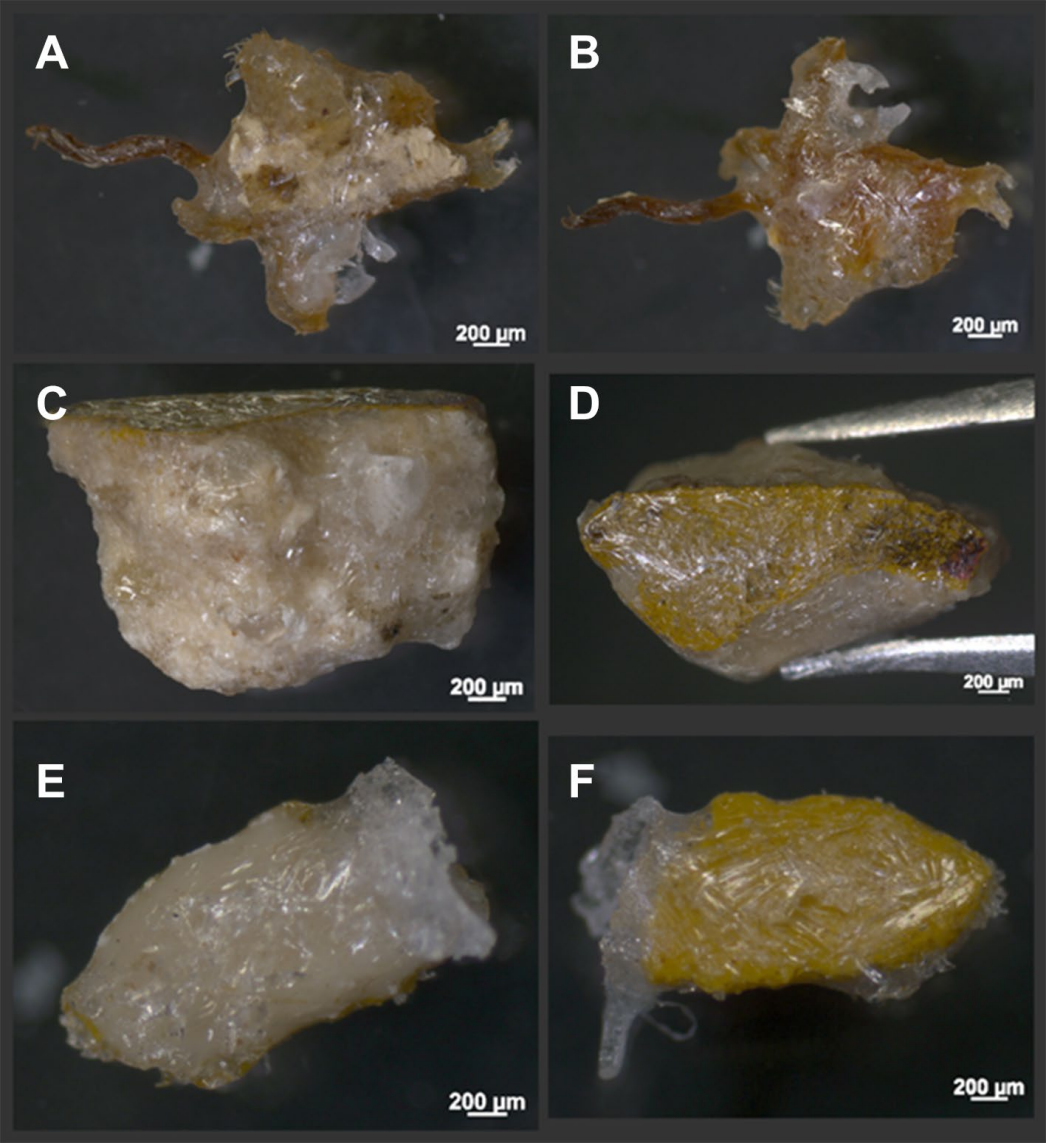
First layer of CDD on the samples; (**A**) RS1, bottom—preparatory layer and (**B**) RS 1, top—coloured layer. One fibre going out of the main part of RS1 is visible on the left part. CDD is not evenly distributed. (**C**) RS2, side view—paint stratigraphy and (**D**) RS2, top—coloured layer. The CDD layer is evenly distributed, but due to the shape of the sample, another CDD layer is needed for a clean extraction from the resin. (**E**) MS1, bottom—preparatory layer and (**F**) MS1, top—coloured layer. Large crystals of CDD reflecting the light are visible, particularly in (**F**).

RS1 (Fig. 1A,B) is a micro-sample (2.5 mm × 1.6 mm × 0.4 mm) from an easel painting on a jute canvas composed of three layers. The top layer is an organic varnish, followed by a green earth layer and finally a preparatory layer of natural chalk.

The RS2 wall painting sample (Fig. 1C,D) is a fresco composed of two layers (2.3 mm × 2.5 mm × 1.4 mm), the top layer is a yellow ochre, and the preparatory layer is a carbonate-sandy mortar bound by calcium carbonate. This sample has the highest porosity, which makes it relatively brittle and sensitive, thus, perfect for this study.

The model sample MS1 (Fig. 1E,F) has two painted layers on a wooden support (2.2 mm × 1 mm × 0.2 mm). This sample consists of a yellow ochre bound with an egg yolk (tempera) as a colour layer, and natural chalk mixed with rabbit skin glue as a preparatory layer. MS1 has low porosity, and the layers can be separated easily. The model sample was cut into several micro-samples smaller than 3 mm.

### Embedding medium—EpoFix

EpoFix (Struers, DK) cold mounting resin was used as the embedding medium. It is a transparent dual-component resin combining epoxy and a hardener. This product was chosen for its low fluorescence^72^, cold curing, easy use, accessibility, low shrinking, and proven applicability for artworks^26^. The resin with no sample inserted yet was introduced in a CitoVac chamber for vacuum impregnation (Struers, DK). This helps remove bubbles in the resin, which reduces the stability and strength of the resin^73^.

### Cyclododecane (CDD)

A crystalline CDD from Sigma-Aldrich (S436402) was acquired for the experiment. The seller did not determine the purity of the material. We used less than half a gram of CDD for the whole study.

#### CDD purity

According to Piotrowski^67^, CDD leaves virtually no traces on the sample, but Caspi^70^ showed that some residues are left occasionally. We decided to verify the purity of our CDD and its possible interaction with the chosen samples.

RS1, RS2 and MS1 were each weighed with a KERN PFB 300-3 scale (1 mg) before being covered with CDD. At different times during the experiment, that lasted up to 122.3 h for a single round, and at the end of it, the samples were weighed (R1/2/3) (Table 1). The same samples were used for each round and covered with CDD repeatedly. Their weight was expected to change if there had been residues of CDD. During the sublimation time, the samples on a glass slide were resting on a hot plate at 30 °C for 6 h. Then, they were installed in a closed cabinet to avoid additional humidity and dust settling on them until the end of the round.

**Table 1:**
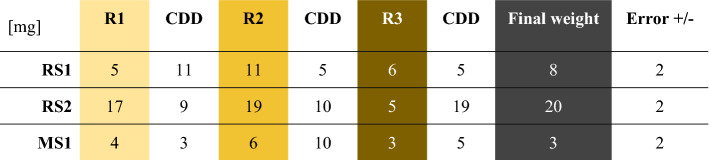
Weight of the samples MS1, RS1 and RS2 before each new round of the CDD sublimation, and CDD added at each round.

An error margin average of +/− 2 mg was calculated based on the averaged difference between the highest and lowest weighted value of each sample.

#### CDD sublimation

The CDD sublimation rate was observed in a fully automated Leica EM ACE 600 high-vacuum coater with a controlled vacuum (atmospheric pressure to ≥ 10^−6^ mbar) and weighed with an Ohaus Adventurer Pro AV264C balance (std dev. of 0.1 mg).

Two CDD grains were chosen. It was decided the procedure would be stopped and the remaining CDD would be weighed when the smaller grain of CDD (0.1 mm in diameter) had disappeared. Weighing CDD in the mid-steps of the increasing vacuum was not considered, as it would have also increased the sublimation rate by the high vacuum changes which do not occur during the real measurements. The two grains were weighed before the procedure (16.2 mg) and fixed on a cover glass with double-sided tape. They were then inserted into the Leica EM ACE 600 coater onto the main platform. The vacuum was increased by steps of 1 mbar and stopped for 1 min at each step, from 104 mbar to 6.7 × 10^−4^ mbar. When the smaller grain was completely sublimed, the experiment was stopped, and the remaining grain was weighed. The visual observation through pictures and a video in real-time allowed to monitor the changes in the sublimation rate.

### Cross-section preparation

The preparation of the cross-sections was started by covering the sample with several layers of a melted CDD (Fig. 1). The micro-sample was not heated, but kept at room temperature to keep a temperature difference between itself and the melted CDD. This quickened the CDD cooling and ensured a more homogeneous layer of large CDD crystals with a length of approximately 200 µm each (Fig. 1). CDD was melted in a glass beaker and kept in a hot-water bath during the process. The micro-sample was dipped into the melted CDD—three times in our case—until the surface was thoroughly covered, and the larger pores were filled by CDD. In Fig. 1, there are visible shape differences between the micro-samples (Fig. 1A–F), which can increase the complexity of the CDD embedding. To simplify the sample extraction without compromising its stability during the SEM measurement, the CDD was elongated and thinned into a triangular shape around the sample. After the sublimation of CDD, the empty triangular shape allowed the insertion of a needle between the sample and the resin to help its extraction, while the thinning of the CDD prevented the sample from moving too much.

**Figure 2:**
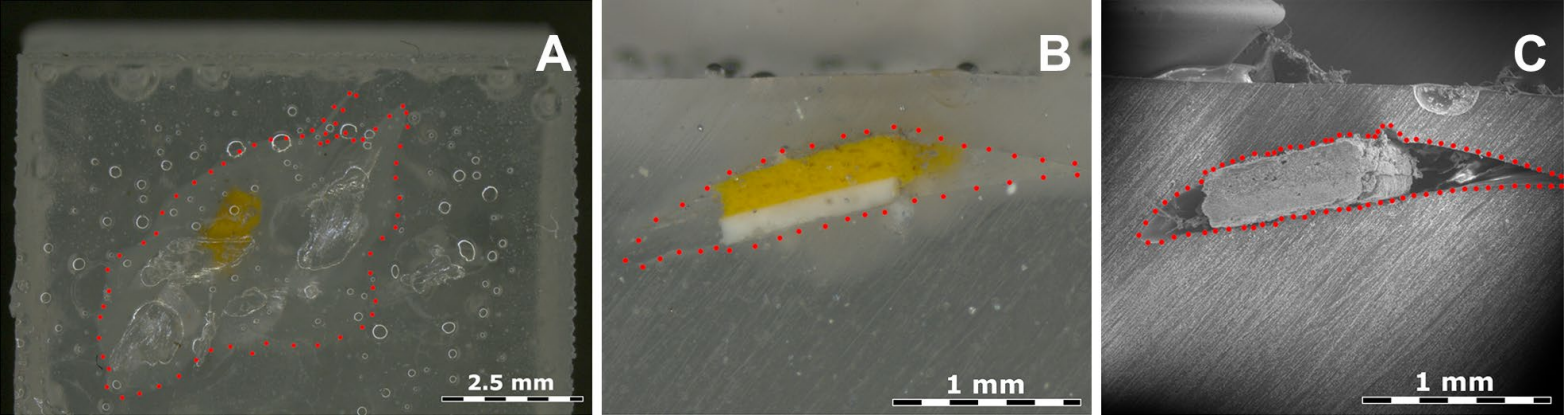
MS1 cross-section embedded in CDD (red dots). (**A**) Optical microscope side view before polishing. (**B**) Optical microscope top view after manual dry polishing, and (**C**) SEM image in LVSTD mode at 10 kV, 30 Pa.

The CDD-covered micro-sample was flat-embedded in the EpoFix and left to cure (Fig. 2A). A silicone ice cube tray of 1 cm × 1 cm × 1 cm was used as a mould, with the first layer of resin already dry before the application of CDD on the sample. The resin protects against sublimation. The cross-section polishing was performed on the same day as the SEM measurement to avoid an early sublimation (Fig. 2B).

The grinding and polishing of the cross-sections was done with a Tegramin-30 device (Struers, DK). SiC foils with grits from 180 to 2000 (grain size from 82 to 10 μm) were used for grinding and polishing cloths in combination with a mixture of diamond suspension and ethanol were used. The automatic wet mode with pressure from 15 to 25 Pa and the manual mode in a wet and dry setting were tested.

Every step from the polishing to the measurement is better done the same day for higher stability of CDD, as it begins to sublime as soon as the resin on top is removed. The sublimation speed depends on the environmental conditions and leaves a void (Fig. 2C).

### Visual examination

The visual description of the micro-sample and cross-section surface was made under the Stemi 2000-C and the Stemi 508 stereo microscopes (both Zeiss, DE) and a Reichert microscope (Reichert Technologies, US), both coupled to an Axiocam ERc 5 s (Zeiss, DE).

The high-resolution imaging was performed in the StAN laboratory of CEITEC-BUT using the MIRA3 XMU scanning electron microscope (Tescan, CZ; high-vacuum 5 mbar to 9 × 10^−5^ mbar). A low-vacuum (0.07–5 mbar) secondary electron detector (LVSTD; Tescan, CZ) was used.

## Results and discussion

### CDD purity determination

**Figure 3:**
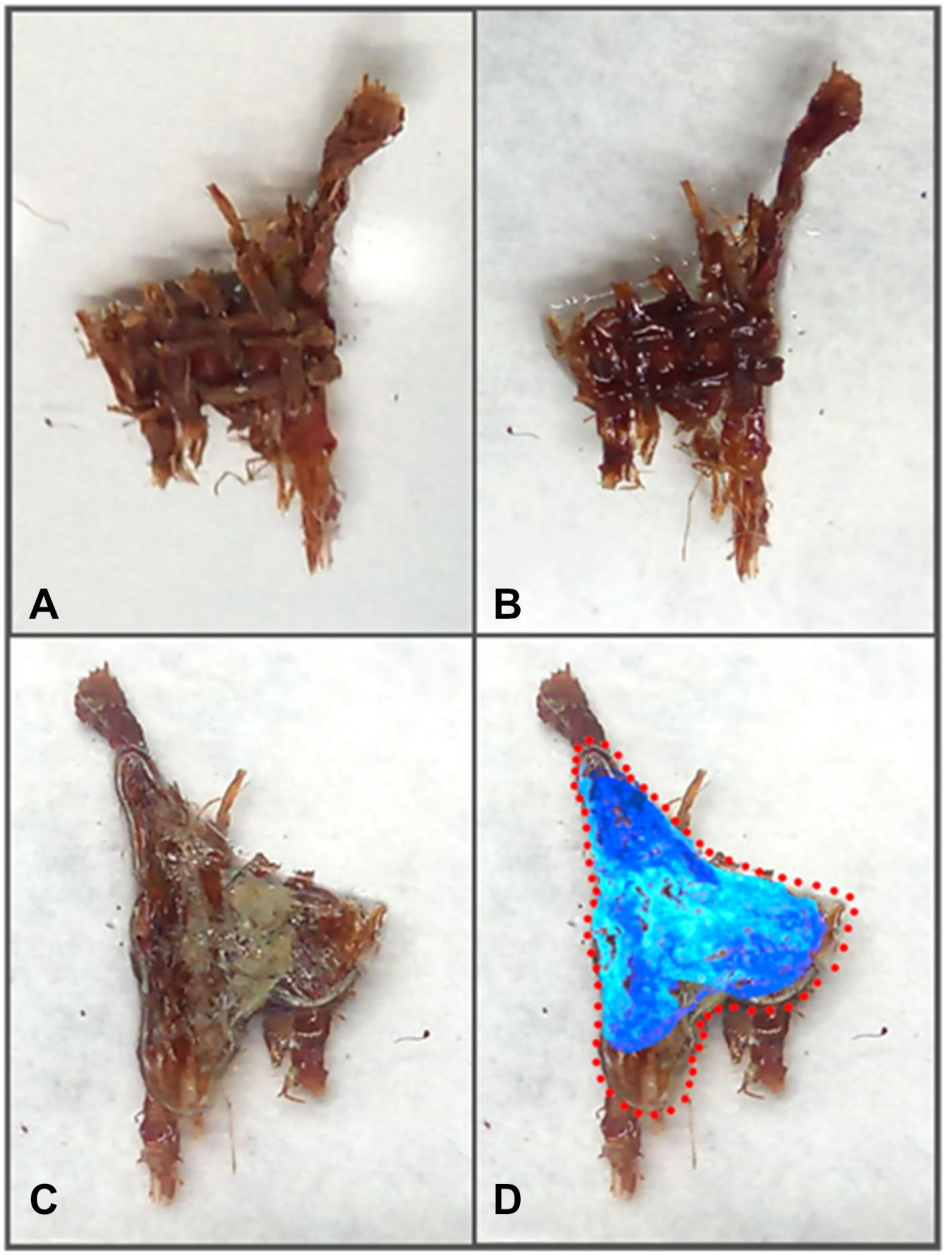
Optical microscope image of RS1 (**A**) before the application of CDD. (**B**) Covered with CDD. (**C**) Through the glass slide where CDD fix the sample onto it. (**D**) CDD delineation (red dots) after its application, and the intensity of the traces left after its sublimation (blue hues). The red dots are based on the visible CDD border of the image (**C**), while the CDD traces after sublimation had to be enhanced by GIMP software from the picture of the glass slide after removing the sample. The blue hues were applied based on the CDD traces to enhance their visualisation and variability. The traces were then added as a mask onto the previous picture to show the difference with the primary CDD layer before sublimation. In the dark blue areas, the traces were clearly visible to the naked eye, whereas they were much more subtle in the light blue areas.

CDD temporarily modifies the strength and visual aspect of the samples, which look glossy and bulkier (Fig. 3A,B). CDD can also be used to fix the sample on the glass slide (Fig. 3C).

We established that sublimation was complete when the samples were not attached to the glass slide anymore. After the sublimation of CDD and the removal of the sample from the glass slide, residues such as a thin delineation and particles were observed. The particles clearly came from the samples, but the delineation seemed to correspond with the CDD pattern, with some shrinkage before its sublimation (Fig. 3D). These kinds of lines appeared on all slides after the CDD sublimation. This confirms the observations of Caspi^70^ that CDD leaves some sort of residue, even though most of it disappears.

**Figure 4:**
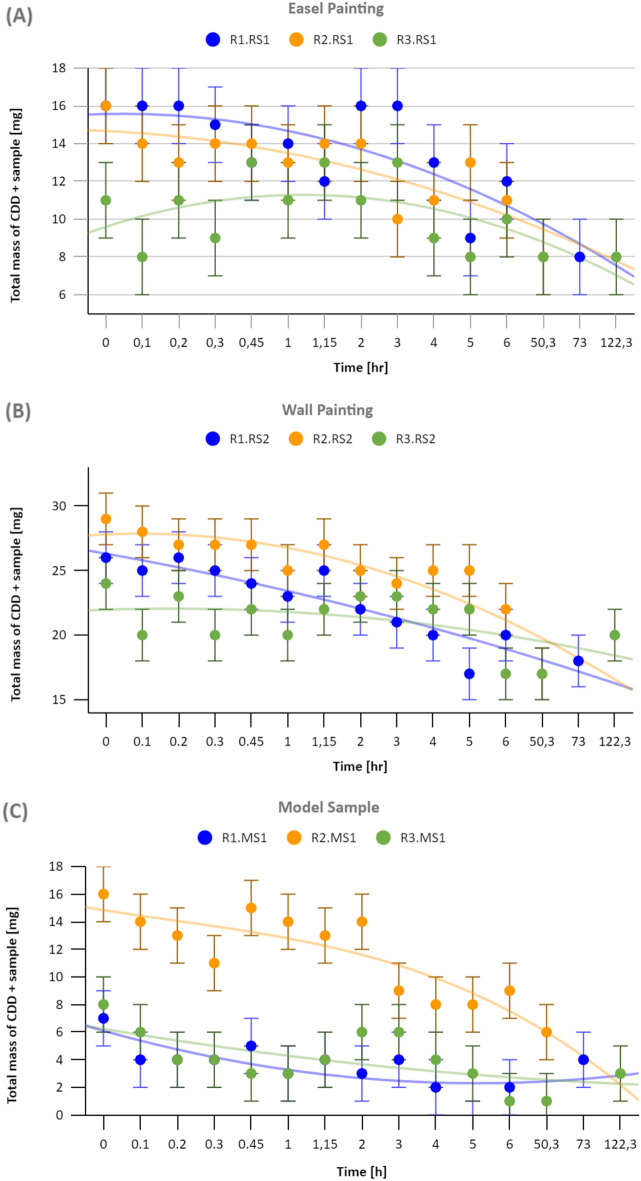
Graphs of the CDD sublimation rate for (**A**) RS1, the painting on canvas, (**B**) RS2, the wall painting, and (**C**) MS1, the model sample. The experiment was reproduced three times on each sample (R1/2/3).

Each graph of Fig. 4 shows the weight changes of a particular sample + CDD in 3 cycles (R1/2/3). The weight of each sample and the applied CDD before each round was calculated with an error margin of ± 2 mg in Table 1. The sublimation of CDD at atmospheric pressure and room temperature (20 °C) began 10 min after its application (Fig. 4). A regular decreasing tendency in the weight can be assessed, which confirms the sublimation of the CDD. However, the experiment conditions have had an impact on the weight. A weight increase (order of 4 mg) was observed between 45 min and 2 h in the case of R2.MS1 (Fig. 4C) and between 2 and 3 h in the case of R3.MS1 and R1.RS1 (Fig. 4A). This increase could occur due to a generally increasing humidity in the room, but in such a case, it should have been similar for all samples, or a local humidity peak occurred (not probable). However, as it was a very low change in weight, an error in the scale balance calibration can be the cause. We also observe rapid point-to-point increase and decrease in the order of 4 mg until 3 h for R3.RS1 (Fig. 4A) and 1h15 for R3.RS2 (Fig. 4B). But, RS2 is the more constant over the 3 rounds (Fig. 4B), while MS1 (Fig. 4C) has almost a perfect overlap between its round 1 and 3, and RS1 (Fig. 4A) has larger random changes. These stronger and quicker changes for RS1 are probably due to the presence of the canvas fibres.

The different CDD absorption capabilities of each sample did not change the general CDD sublimation rate according to the final weights (Tab. 1), even though the canvas fibres of RS1 (Figs. 3A,B, 4A) showed a higher disposition to absorb CDD. Compared to RS2 and MS1, it resulted in a thinner layer of CDD around this particular sample with the same amount of CDD applied.

The decreasing weight tendency of all samples confirms the CDD sublimation, however, the final weight is not 0 g, thus, some residues are present (Fig. 4; Table 1).

### CDD sublimation rate in a vacuum

At atmospheric pressure, CDD usually sublimes in a matter of hours to weeks, depending on the application process, the thickness of the layer and the surrounding environment (temperature, humidity). The sublimation rate will therefore increase or decrease. At the beginning of our experiment, it was observed that CDD in a high vacuum sublimed in less than 5 min. To reduce the sublimation rate, the vacuum in the SEM chamber should be carefully selected. Therefore, it was decided to explore the sublimation of CDD under a vacuum before finalising the embedding procedure, because of its direct impact on the stability of the micro-sample during SEM observations.

**Figure 5:**
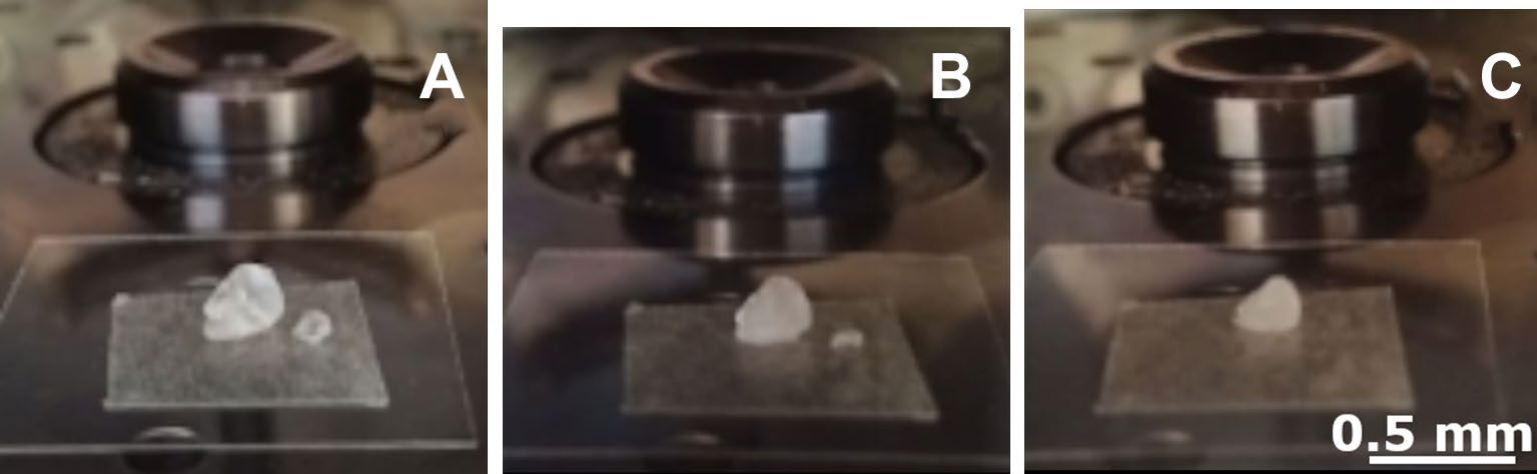
Inside the vacuum chamber of the Leica coater, with the CDD grains prepared for the sublimation rate control. (**A**) The large (left) and the small (right) grains of CDD at atmospheric pressure (104 mbar) before the experiment. (**B**) The two grains at 1.6 × 10^−1^ mbar. (**C**) The remaining large grain at the end of the procedure stopped at 6.7 × 10^−4^ mbar when the small grain completely disappeared.

The CDD grains were placed on the vacuum coater platform (Fig. 5A). Most of the CDD sublimation happened between 1.6 × 10^−1^ mbar and 8.7 × 10^−2^ mbar (Fig. 5B). The smaller CDD grain was completely sublimed at 6.7 × 10^−4^ mbar (Fig. 5C). There was no visible sublimation between 8.7 × 10^−2^ mbar and 6.7 × 10^−4^ mbar (Fig. 5C). Changes unnoticeable to the naked eye might have occurred, or CDD might have stabilised at this pressure.

When the experiment was stopped (Fig. 5C), the remaining CDD weighed 4.8 mg. The total CDD weight loss was 11.4 mg. This loss can have a noticeable impact on the stability of the micro-sample in the cross-section during a measurement. The surface of the micro-sample can tilt due to the sublimation, depending on the distribution of CDD around it. According to our experiment, the vacuum should be lower than 1.6 × 10^−1^ mbar. However, even in this case, the CDD sublimation will continue at a higher speed than at the atmospheric pressure. Use of the lowest vacuum possible is recommended.

### Sample extraction from the cross-section

CDD crystallises in big needles around the micro-samples (Fig. 1), and the shape of the CDD layer should be chosen not to disturb the stability of the micro-sample even after its sublimation. The CDD layer thickness was around 0.01 mm on the flat surface and up to 0.5 mm from the borders of the micro-samples (Fig. 2C). The opaqueness of the layer increases with its thickness. This can be problematic for positioning the micro-sample if the stratigraphy is not visible (Fig. 1B). For this reason, the layer should be thick enough to fully cover and support the micro-sample, but thin enough to enable recognition of its external features. The need to use a microscope is therefore emphasised in this part of the preparation, especially when the samples are smaller than the ones from our study. Regarding such smaller samples, a thick layer of CDD can be first applied, and then cut and reshaped around the micro-sample using a heated needle.

The extraction was simplified by forming CDD into a triangular shape around the micro-sample. This created a void after the CDD sublimation, while keeping the micro-sample stable within the cross-section (Fig. 2A,B) even after the CDD sublimation (the micro-sample was held in place when the cross-section was turned upside-down and shaken).

**Figure 6:**
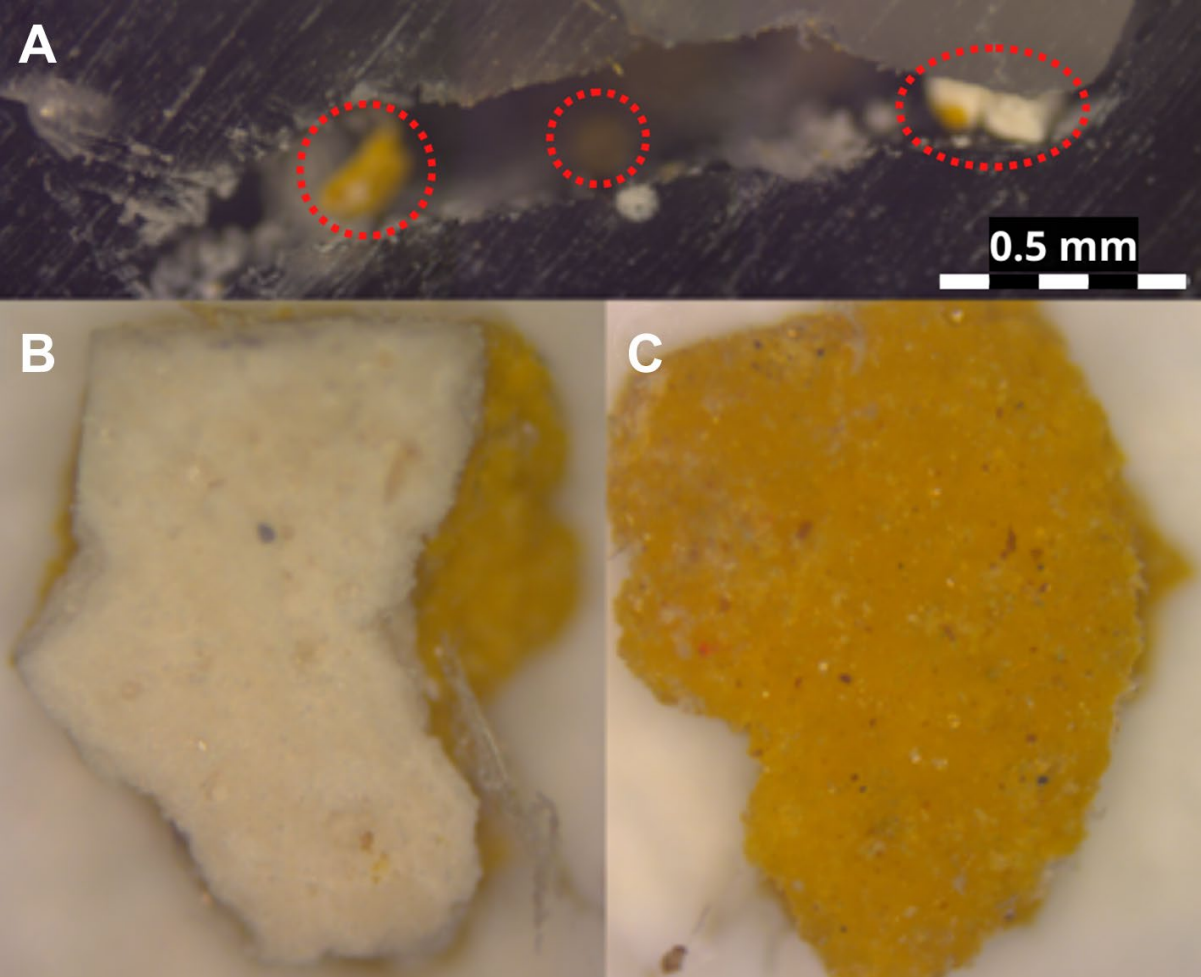
Light microscopy images of MS1: (**A**) Resin with the residues (circled) of the micro-sample after its extraction. (**B**) Extracted MS1, bottom and (**C**) MS1, top.

The created void (Fig. 2C) was just large enough for a needle tip to fit in. The micro-sample was extracted from the cross-section by gently pushing it out from the bottom using a flexible needle. Small parts of the sample broke away during the extraction (Fig. 6A). It might be caused by an insufficient layer of CDD on weak parts of the sample, such as the edges or unconsolidated layers. In Fig. 6B, the preparatory and the paint layers are not properly bound, which creates weak points that need more protection to avoid breakages. As it can be seen on the right side of the micro-sample, where only the yellow-coloured layer is visible.

The risk of losing a small amount of the sample must be considered. In our case, this loss concerned a few tiny parts of the micro-sample which stayed in the resin (Fig. 6A), probably due to an insufficient CDD layer or the mechanical extraction. The volume of the micro-sample was 0.44 mm3 and the one of the residues in the resin was approximately 0.018 mm3. The loss is approximately 4% of the micro-sample. As the embedded parts of a sample would not be otherwise accessible, we believe this loss can be considered negligible, if it was possible to extract more than 90% of the sample’s original volume. The extracted sample showed no signs of residual resin, CDD or changes in colour or texture (Fig. 6B, C).

## Conclusion

This study was focused on enhancing the widely used preparation of cross-sections for painting micro-samples, by exploring the possibility of their later extraction. Although the extraction is not always necessary, the need to re-analyse the bulk of the sample, inaccessible from the polished surface of the cross-section, or the degradation of the embedding material, is among the reasons for this feasibility study.

The application process of CDD, which is usually used as a protective layer to prevent sample contamination by the embedding medium, was adjusted to simplify the extraction of the micro-sample. The CDD protective layer was formed into a triangular shape around the micro-sample to create specifically shaped voids, but also to keep the stability of the sample inside the cross-section after the sublimation of the CDD. The voids were then used to insert a flexible needle to gently extract the sample. A loss of the sample material occurred, however, since its total volume was about 4% of the original volume of the sample, it was considered negligible. No other changes in the micro-sample were observed.

The CDD purity and its sublimation rate in the vacuum were also studied to ensure the safe use of CDD in the field of cultural heritage. The sublimation rate was relatively low up to 1.6 × 10^−1^ mbar, which kept the stability of the cross-section during a low vacuum or environmental SEM measurement, where the vacuum goes from the atmospheric pressure up to 1.33 mbar or 30 mbar respectively. Nevertheless, the sublimation rate was still higher than at atmospheric pressure even in this range. When CDD sublimes, it leaves negligible traces, which allows for performing other analyses requiring polished surfaces or uncontaminated micro-samples.

Until the polishing of the cross-section, the CDD layer protects the micro-sample. Even after polishing, the whole cross-section can be stored and the sample extracted at any time, while keeping the advantages of the cross-section preparation. Sealing the polished cross-section again by an additional layer of CDD and resin might be suggested as further protection of the micro-sample, but such a solution would have to be explored in further studies. Although in such a configuration, the oxidation of metallic and organic components present in the sample could also be avoided without compromising the material composition and structure of the sample.

The suggested procedure is simple and uses materials that most laboratories are equipped with. Although CDD can be considered expensive, the quantity used in this study was less than half a gram. Therefore, its cost is not a limiting factor for such a purpose. Moreover, considering the importance and uniqueness of the micro-samples, this study proved that CDD is an asset that enables their extraction and avoids their contamination, increasing the number of possible analyses. The use of a protective layer, such as CDD, regarding its shape, which enables a later extraction of the sample, should become a routine part of the preparation of cross-sections of micro-samples from unique cultural heritage objects, such as paintings.

## Data Availability

All data generated or analysed during this study are included in this published article.
